# CRISPR/Cas9-editing of *KISS1* to generate pigs with hypogonadotropic hypogonadism as a castration free trait

**DOI:** 10.3389/fgene.2022.1078991

**Published:** 2023-01-04

**Authors:** Julio M. Flórez, Kyra Martins, Staci Solin, Jonathan R. Bostrom, Paula Rodríguez-Villamil, Felipe Ongaratto, Sabreena A. Larson, Uyanga Ganbaatar, Alexander W. Coutts, Doug Kern, Thomas W. Murphy, Eui-Soo Kim, Daniel F. Carlson, Abe Huisman, Tad S. Sonstegard, Clay A. Lents

**Affiliations:** ^1^ Acceligen Inc., Eagan, MN, United States; ^2^ Department of Preventive Veterinary Medicine and Animal Reproduction, School of Agricultural and Veterinarian Sciences, São Paulo State University (Unesp), Jaboticabal, Brazil; ^3^ Recombinetics Inc., Eagan, MN, United States; ^4^ USDA, ARS, U.S. Meat Animal Research Center, Clay Center, NE, United States; ^5^ Hypor, Hendrix Genetics, Boxmeer, Netherlands

**Keywords:** kisspeptin, pig puberty, animal welfare, boar taint, embryo editing, homology-directed repair, knockout

## Abstract

**Introduction:** Most male pigs are surgically castrated to avoid puberty-derived boar taint and aggressiveness. However, this surgical intervention represents a welfare concern in swine production. Disrupting porcine *KISS1* is hypothesized to delay or abolish puberty by inducing variable hypogonadotropism and thus preventing the need for castration.

**Methods:** To test this hypothesis, we generated the first *KISS1*-edited large animal using CRISPR/Cas9-ribonucleoproteins and single-stranded donor oligonucleotides. The targeted region preceded the sequence encoding a conserved core motif of kisspeptin. Genome editors were intracytoplasmically injected into 684 swine zygotes and transferred to 19 hormonally synchronized surrogate sows. In nine litters, 49 American Yorkshire and 20 Duroc liveborn piglets were naturally farrowed.

**Results:** Thirty-five of these pigs bore *KISS1*-disruptive alleles ranging in frequency from 5% to 97% and did not phenotypically differ from their wild-type counterparts. In contrast, four *KISS1-*edited pigs (two boars and two gilts) with disruptive allele frequencies of 96% and 100% demonstrated full hypogonadotropism, infantile reproductive tracts, and failed to reach sexual maturity. Change in body weight during development was unaffected by editing *KISS1*. Founder pigs partially carrying *KISS1*-disruptive alleles were bred resulting in a total of 53 *KISS1* ^+/+^, 60 *KISS1* ^+/−^, and 34 *KISS1* ^−/−^ F1 liveborn piglets, confirming germline transmission.

**Discussion:** Results demonstrate that a high proportion of *KISS1* alleles in pigs must be disrupted before variation in gonadotropin secretion is observed, suggesting that even a small amount of kisspeptin ligand is sufficient to confer proper sexual development and puberty in pigs. Follow-on studies will evaluate fertility restoration in *KISS1* KO breeding stock to fully realize the potential of KISS1 gene edits to eliminate the need for surgical castration.

## 1 Introduction

Surgical castration is routinely implemented in male piglets destined for pork production to prevent the development of boar taint and reduce androgen-driven behaviors (e.g., aggression and mounting) that increase the risk of injuries (Rault et al., 2011). Boar taint is a an off-odor and strong flavor found in meat from intact male pigs that consumers find unacceptable. Surgical castration is a welfare concern because it is considered painful and methods for effective analgesia are largely unavailable (Rault et al., 2011). Some alternatives to this management procedure are commercially available, such as immunization against gonadotropin-releasing hormone (GnRH) (Dunshea et al., 2001), but there are a number of constraints that limit their use (Bonneau and Weiler, 2019; Squires et al., 2020); and the goal of eliminating castration has not proven feasible (Backus et al., 2018).

We sought a genetic mechanism to avoid the need for surgical castration. Editing a gene to block sexual maturation was a promising approach as male pigs that remain prepubertal are not expected to develop boar taint and aggressive behavior. We selected the kisspeptin system based on its conserved role in initiating mammalian puberty (Lents, 2019; Uenoyama et al., 2019; Sobrino et al., 2022). Kisspeptin is a peptide encoded by the highly conserved KISS1 gene, that stimulates the release of GnRH and secretion of gonadotropins (Lents, 2019). Mutations in the kisspeptin receptor gene (*KISS1R*) result in hypogonadotropic hypogonadism (HH) and insufficient sexual maturity in humans (de Roux et al., 2003; Seminara et al., 2003; Semple et al., 2005). Similarly, knocking out either Kiss1 (d’Anglemont et al., 2007; Lapatto et al., 2007; Uenoyama et al., 2015; Ikegami et al., 2020) or Kiss1r (Funes et al., 2003; Seminara et al., 2003; Lapatto et al., 2007) genes in laboratory rodents results in pubertal failure and infertility owing to HH. Hypogonadotropic hypogonadism was also induced in pigs by Sonstegard et al. (2016), Sonstegard et al. (2017), who developed the first large animal model of impaired kisspeptin system when they knocked out *KISS1R* in pigs using TALENs (Tan et al., 2013), demonstrating that kisspeptin signaling is vital for sexual maturation of boars.

Humans, mice, and pigs with impaired KISS1/KISS1R genes have responded to exogenous GnRH, gonadotropins (Seminara et al., 2003; Sonstegard et al., 2017), or kisspeptin analogs (d’Anglemont et al., 2007; Lapatto et al., 2007), although some of these approaches only partially reversed the *KISS1R* KO phenotype in boars (Sonstegard et al., 2017). We hypothesized that *KISS1* KO pigs would be phenocopies of *KISS1R* KO pigs, as observed in some *Kiss1* KO mice (Lapatto et al., 2007). However, editing *KISS1* should have the advantage of making fertility rescue less challenging because the ligand can be given exogenously. Therefore, *KISS1* KO pigs were generated to test the hypothesis that the kisspeptin ligand is essential for sexual maturity in swine. This was accomplished by microinjecting CRISPR/Cas9-ribonucleoproteins (RNPs) and homology-directed repair (HDR) templates into *in vivo* fertilized porcine zygotes, to create mosaic founder (F0) pigs harboring *KISS1*-disruptive alleles. Pigs with partial disruption of this gene were used to produce piglets carrying *KISS1*-edited alleles without mosaicism.

## 2 Results

### 2.1 Generation of *KISS1*-edited pigs *via* zygote microinjection

**FIGURE 1:**
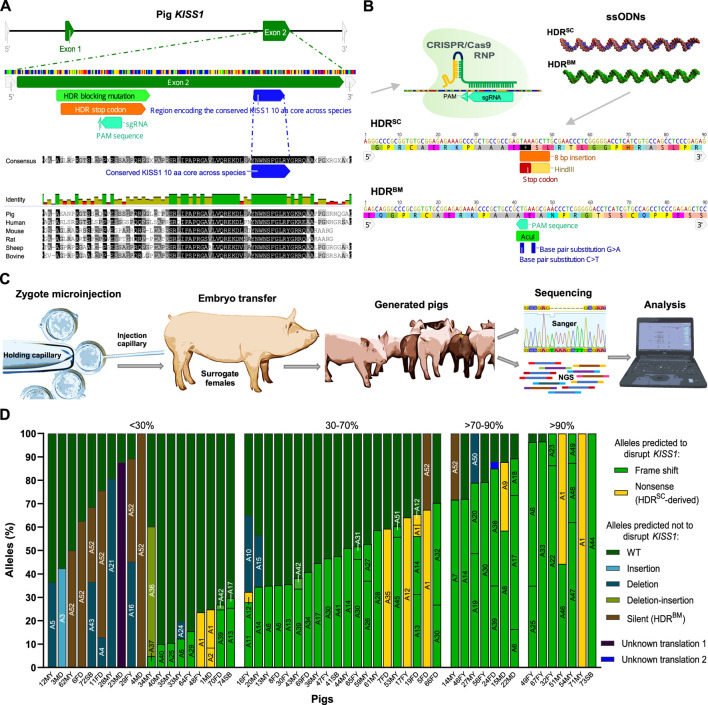
Generation and genotyping of *KISS1*-edited pigs *via* zygote injection of CRISPR/Cas9 and single-stranded donor oligonucleotides (ssODNs). **(A)** Graphics depicting the genomic structure of *S. scrofa KISS1*, the gene sites bound by the designed sgRNA as well as the homology-directed repair (HDR) templates, and encoding the conserved amino acid core of this protein. At the lower part, it is shown the kisspeptin sequence alignment among different mammalian species in the region flanking the highly conserved core of this protein. **(B)** Schematic representation of CRISPR/Cas9 RNPs targeting *KISS1* besides both ssODNs, and sequences of these HDR templates used to introduce a stop codon and the HindIII restriction site (HDR^SC^) as well as synonymous SNPs, to change the PAM sequence, to reduce recutting and generate the AcuI restriction site (HDR^BM^). **(C)** Schematic of the intracytoplasmic microinjection of CRISPR components and ssODNs into porcine zygotes, transfer of injected embryos, obtention of pigs, and their genotyping for the targeted locus. **(D)** Bar graph showing gene-edited pigs grouped according to their *KISS1*-disruptive editing percentages and depicting the allele names and percentages in each individual. In the *X*-axis are the pig IDs, in which F stands for female, M for male, Y for Yorkshire, D for Duroc, and SB for stillborn.

To introduce loss-of-function mutations in swine *KISS1*, a single guide RNA (sgRNA) was designed to target the second exon of this gene, upstream of the sequence encoding the highly conserved KISS1 10-amino acid (aa) core (Figure 1A). The designed sgRNA and Cas9 proteins were co-injected into porcine zygotes with two HDR templates, which separately contained a stop codon (HDR^SC^) and a silent blocking mutation (HDR^BM^). These single-stranded donor oligonucleotides (ssODNs) were paired to increase the odds of generating pigs with edits capable of disrupting *KISS1* (HDR^SC^) and/or maintaining *KISS1* function (HDR^BM^; Figures 1B, C). A total of 684 zygotes were injected and transferred into 19 recipient sows. After full-term pregnancies, 69 liveborn (35 males and 34 females) and five stillborn piglets were farrowed in nine litters (Supplementary Table S1).

Analyses of DNA sequences from tail tissues revealed that 53 (72%) piglets were edited, and 18 of these (24%) bore HDR-mediated edits (Table 1). At least 22 pigs bore multiple (>2) alleles. In pigs with two different alleles, 24 pigs had one wild type (WT) allele while four animals had two different editing-derived alleles. AmpSeq indicated that most of the pigs bearing two alleles were mosaic instead of heterozygous (monoallelic edit) or compound heterozygous (different biallelic edits). Three pigs (4MD, 71MY, and 73SB) presented identical biallelic edits, of which there was one pig for each HDR-intended mutation (Figure 1D).

**TABLE 1:** Genotype summary of pigs produced *via* microinjection into zygotes of CRISPR/Cas9 RNPs and ssODNs intending to disrupt *KISS1*.

Editing efficiency (number of pigs)							Number of generated pigs according to their editing percent						
Total	NHEJ	HDR-derived				*KISS1* disruptive	Editing percent ranges	Total			*KISS1* disruptive		
		ssODN	Total	Perf.	Imp.			Total born	Liveborn	Alive at weaning	Total born	Liveborn	Alive at weaning
72% (53/74)	59% (44/74)	HDR^SC^	14% (10/74)	8% (6/74)	8% (6/74)	58% (43/74)	WT/0%^a^	21	20	16	31	29	24
							<30%	7	7	5	9	8	6
							30%–70%	26	23	21	20	19	17
		HDR^BM^	12% (9/74)	11% (8/74)	1% (1/74)		>70%–90%	9	9	7	7	7	6
							>90%	11	10	9	7	6	5

NHEJ, non-homologous end joining repair; ssODN, single-stranded donor oligonucleotides; HDR, homology-directed repair; HDR^SC^, HDR^stop codon^; HDR^BM^, HDR^blocking mutations^; Perf., perfect HDR-derived edit; Imp., imperfect HDR-derived edit.

^a^ WT for “Total” and 0% for “*KISS1* disruptive.”

Detected indels ranged in size from 1 to 68 bp for insertions and 1 to 211 bp for deletions (Supplementary Figure S1), whereas larger indel alleles would not be detected by our AmpSeq assay. Predicted translations indicate that five and 35 alleles contained non-sense and frameshift mutations, respectively. These alleles were predicted to disrupt the KISS1 aa core (Supplementary Figure S2) and were carried by 43 pigs at different frequencies. The percentage of reads attributed to gene-editing-derived alleles in a pig’s genotype was defined as editing percent. In all, 27 animals were generated with *KISS1*-disruptive alleles between 30% and 90%, with seven pigs having a mosaicism level greater than 90% (Figure 1D).

### 2.2 Phenotypic analysis

**FIGURE 2:**
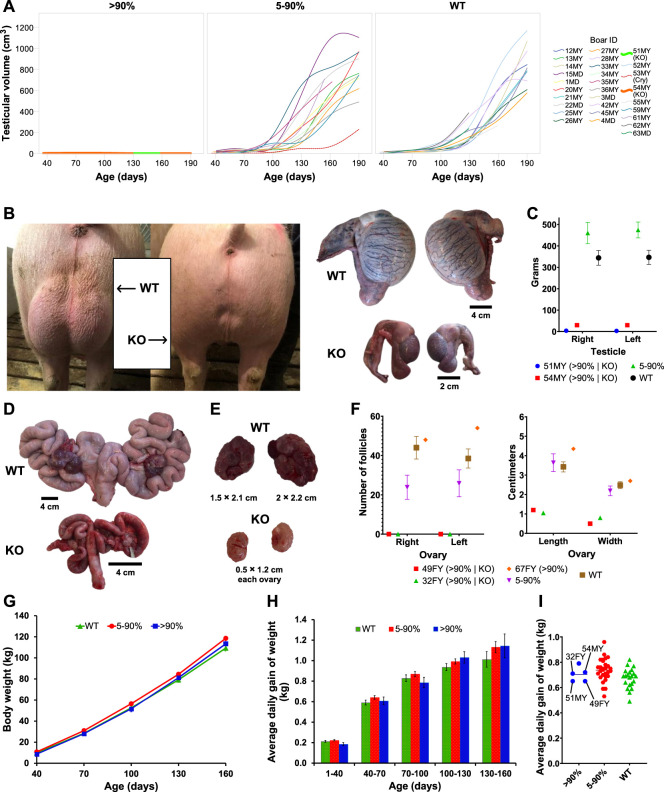
Reproductive organ phenotyping and growth-related traits of *KISS1*-edited pigs. **(A)** Representation of individual testicular volumes collected across different time points, where each panel corresponds to a *KISS1*-disruption group. “Cry” stands for cryptorchid. **(B)** *In vivo* and *ex vivo* comparison of representative WT and *KISS1* KO testicles. **(C)** Comparison of the testicular weight of WT and *KISS1-*edited boars. **(D,E)** Comparison of representative WT and *KISS1* KO gilt reproductive tracts **(D)** and ovaries **(E)**. **(F)** Follicle count and measurements of ovaries. In **(C)** and **(F)**, values are individually represented for pigs with *KISS1*-disruptive editing percentages above 90% and as means for the other groups. **(G,H)** Body weights (LSmeans ± SE; age, *p* < .001) **(G)** and average daily gain of weights (LS means ± SE; birth weight, *p* < .001) **(H)** across the evaluated time points (±4 days) and periods, respectively. **(I)** Scatter plot of total average daily gain of weight (i.e., from birth to 160 days of age) with lines indicating the *KISS1* KO pigs. In **(G–I)**, results are grouped by the *KISS1*-disruptive editing percent.

Four pigs (two boars, two gilts) displayed phenotypic characteristics consistent with hypogonadism. Testicular weight and size were dramatically reduced in two boars (51MY and 54MY) at 8 ± 0.6 months of age compared to control animals (Figures 2A–C; Supplementary Table S2). *KISS1* WT alleles were absent in both of these two boars. In all other boars, typical variation in testicular volume throughout development was observed, and the variation was not related to the extent to which pig’s *KISS1* was disrupted (Figure 2A). The *KISS1* KO gilts had *KISS1*-disruptive allele frequencies of 96% and 100% (49FY and 32FY, respectively) with considerably smaller ovaries and visual absence of surface follicles. The uteri of these *KISS1* KO gilts were underdeveloped when compared to age-matched controls (Figures 2D–F; Supplementary Table S3). These findings alongside behavioral observations and hormonal data (Supplementary Figure S3) suggested that the *KISS1* KO pigs failed to become pubertal. The other *KISS1*-edited and WT pigs were considered sexually mature as they displayed genital sizes and external characteristics typical of boars and gilts that advanced through puberty, which is consistent with the hormonal profiles of these animals. Additionally, mosaic F0 pigs that were mated exhibited sexual behaviors, gamete production, and fertility.

Piglet birth weight was unaffected by sex, *KISS1*-disruption group, and their interaction (*p* ≥ .55). The main effect of age was the only fixed effect to influence body weight (*p* < .001), but average daily gain was influenced by age and piglet birth weight (*p* < .001). There was no significant interactive effect of age × *KISS1*-disruption group for body weight or average daily gain (*p* ≥ .33; Figures 2G, H). Body weight was lower (*p* = .04) in WT than 5%–90% *KISS1*-disruptive pigs at 160 days, but no *KISS1*-disruption groups differed in body weight or average daily gain at any age category evaluated (*p* ≥ .07). In this study, the pigs considered as WT were those that carried no *KISS1*-disruptive alleles, see descriptive statistics in Supplementary Table S4. Boar 15MD was classified in the group >90% *KISS1*-disruption based on initial sequencing, but his phenotype was remarkably similar to an unedited or partially mosaic boar (<90%). Resequencing revealed him to have 88% of *KISS1*-disruptive allele frequency instead of 95% as originally determined. Reclassification of this boar did not alter statistical significances or interpretation of the results. It cannot be ruled out that similar cases may have occurred. Nevertheless, no further inconsistencies were observed between genotype and expected phenotype aside from pig 67FY. This gilt had a *KISS1*-disruptive allele frequency of 97% but had no phenotypic abnormalities.

### 2.3 Testicular immunohistology

**FIGURE 3:**
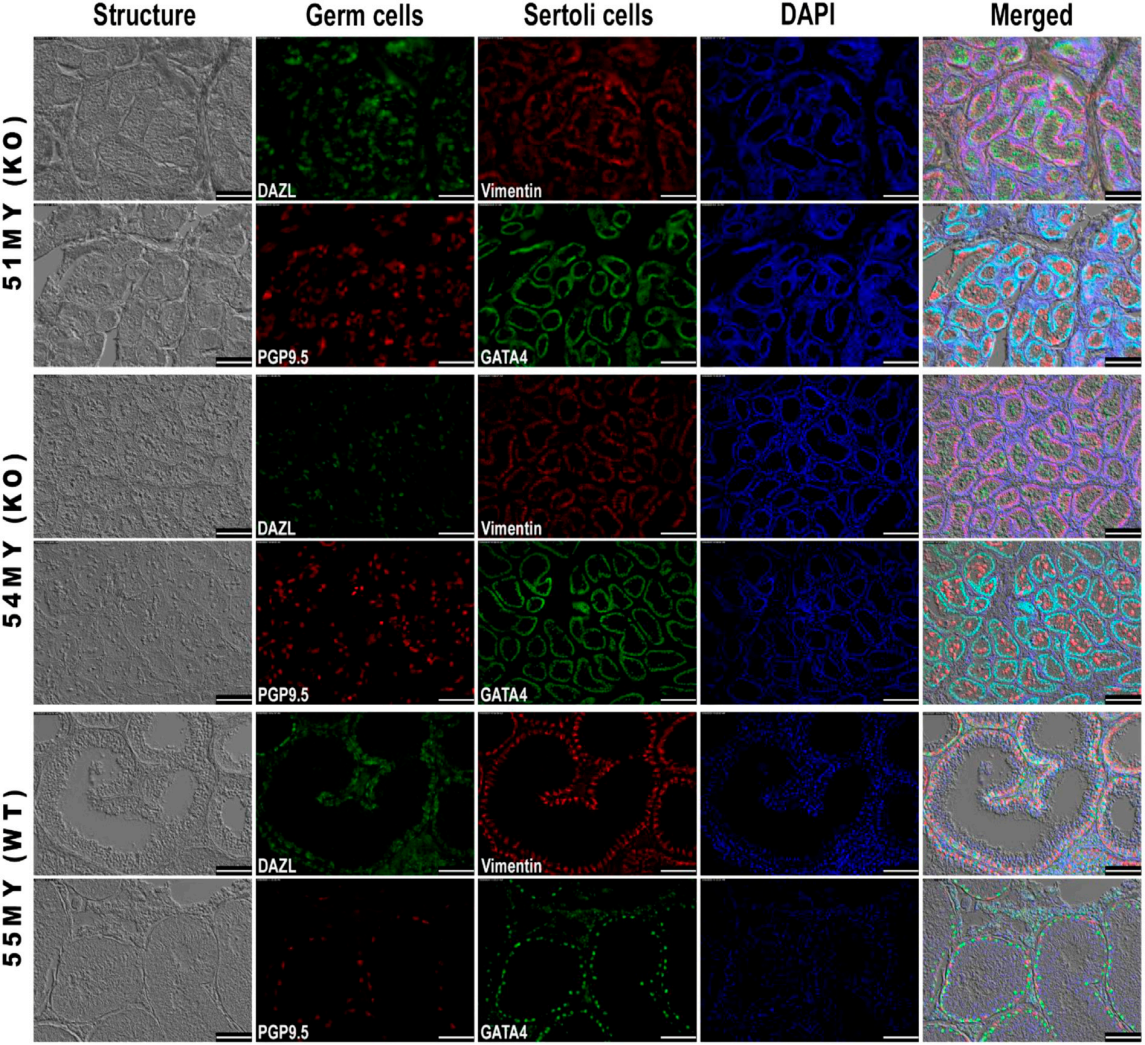
Immunohistochemical analysis of germ and Sertoli cell markers in seminiferous tubules from WT and *KISS1* KO pigs. Boars were 8 months old. DAPI was applied as nuclear staining and is represented in blue fluorescence. Combined images of structure, targeted proteins, and nuclei are shown in the last column. Scale bar = 100 µm.

*KISS1* KO and WT boars presented an architecture of seminiferous tubules that looked similar. The main difference between these two groups of 8-month-old pigs was related to the size of the observed structures, which were smaller in kisspeptin deficient boars. In the *KISS1* KO group, there were no visible lumina and Sertoli cells were arrayed along the parietal edge of these small seminiferous tubules. Germ cells were located both in the adluminal area and toward the edge of the tubules while no spermatids were observed. In the WT boar, the germ cells had all homed to the basement membrane of the germinal epithelium, with none in the seminiferous tubule lumen; germ cells and Sertoli cells were mixed lining the tubule. DAPI staining revealed the presence of cells across the germinal epithelium and adluminal area only in the WT boar, and some sperm tails and elongated spermatids were observed. This indicates that, in this animal, germ cells were undergoing differentiation to eventually become spermatozoa (Figure 3).

### 2.4 Hormonal profiling

**FIGURE 4:**
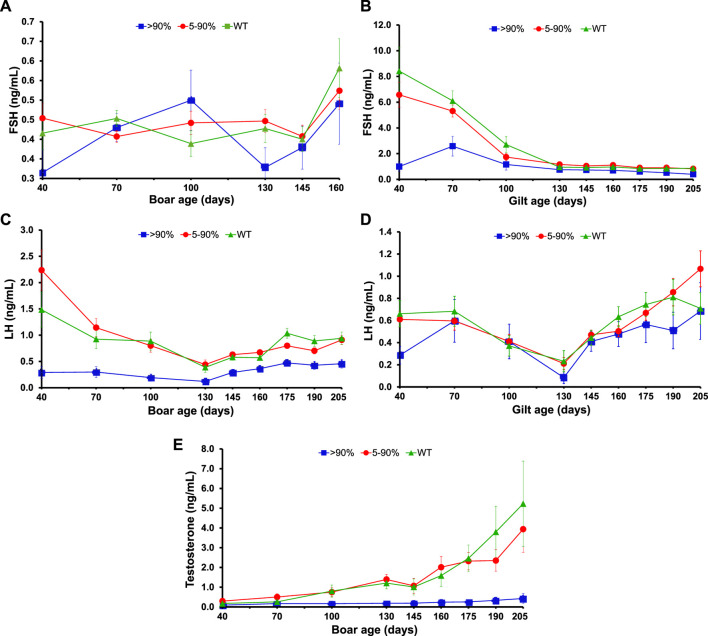
Hormone profiles of *KISS1*-edited pigs. **(A–D)** Serum concentration (LSmeans ± SE) in boars and gilts of FSH [**(A)** and **(B)**, respectively] and LH [**(C)** and **(D)**, respectively] during development by *KISS1*-disruption group. **(E)** Developmental change in serum concentrations (LS means ± SE) of testosterone in boars according to the *KISS1*-disruptive editing percent.

Hormone measurements were conducted every 4 weeks beginning when pigs were 40 days old and halved to a fortnight frequency from 130 to 280 days of age. Only follicle-stimulating hormone (FSH) of gilts showed an interaction of age with *KISS1*-disruptive editing percent (*p* = .05), but least-squares means of this interactive effect are displayed for all hormones in Figure 4 to describe patterns over time. Gilt FSH was lower for the >90% *KISS1*-disruption group than all others at 40 and 70 days of age (*p* ≤ .05; Figure 4B), but no differences between groups were observed thereafter (*p* ≥ .20). Serum concentrations of boar FSH were not affected by *KISS1*-disruptive editing percent (*p* = .39; Figure 4A). All hormones, except for boar FSH (*p* = .07), were influenced by the main effect of age (*p* < .001). Overall, boars with *KISS1*-disruptive allele frequency >90% had less (*p* < .001) serum luteinizing hormone (LH) and testosterone during all ages of development compared with other groups, which did not differ from one another (*p* ≥ .91; Figures 4C, E). Concentrations of LH in gilts with *KISS1*-disruption >90% were numerically less than in other groups, but this did not reach significance (*p* = .07; Figure 4D). Individual concentrations of serum hormones for some pigs belonging to the group with *KISS1*-disruptive allele frequency >90% are shown in Supplementary Figure S3.

### 2.5 F0-to-F1 transmission of *KISS1*-edited alleles

**FIGURE 5:**
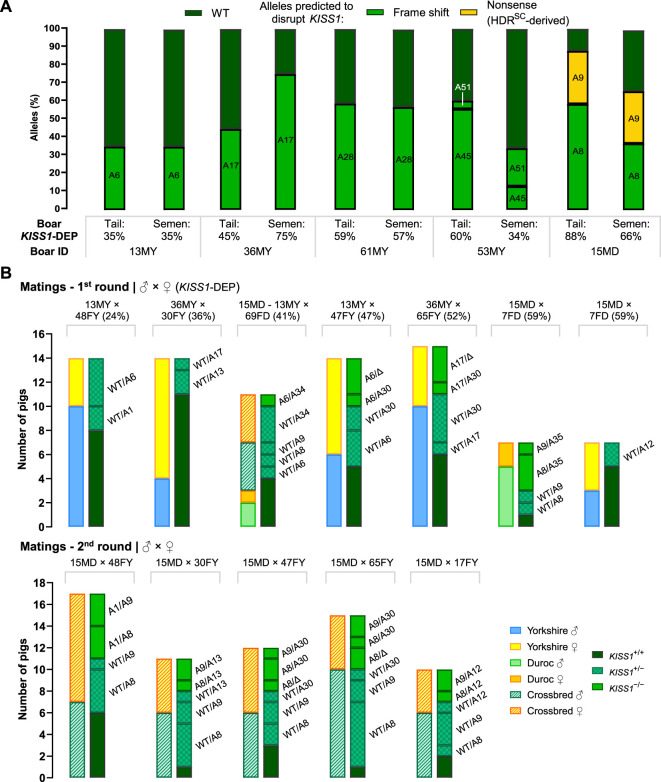
Confirmation of *KISS1*-edited allele transmission. **(A)** *KISS1* allele frequencies of mosaic F0 boar candidates for breeding, calculated using DNA from tail tissue and spermatozoa. **(B)** Inherited *KISS1* alleles by F1 piglets, and the number of genotyped pigs in each litter according to their sex-breed (first bar) or genotype category (second bar). “DEP” stands for disruptive editing percent and “Δ” means deletion.

Intergenerational transmission of the *KISS1*-mediated castration free trait is required to provide a more effective alternative to end surgical castration. Semen from five F0 boars (13MY, 36MY, 61MY, 53MY, and 15MD) with *KISS1*-disruptive allele frequencies between 35% and 88%, was collected for analysis (Supplementary Table S5). *KISS1* gene edits in sperm DNA were evaluated by NGS and compared to results obtained from the tail samples. Each mutant allele detected in the tail samples could be found in the sperm DNA, confirming germline transmission. However, apart from 13MY and 61MY, there was discordance ranging from 16% to 43% between the allele frequencies observed in the tail versus those in sperm DNA (Figure 5).

Three F0 boars and 10 F0 gilts were selected for mating based highest levels of *KISS1* disruption under 90 percent and having the lowest inbreeding coefficient. From the first matings, seven litters were farrowed (Figure 5) with an average of 12.9 ± 1.6 liveborn piglets per litter, for a total of 90 liveborn F1 piglets. The genotype distribution was, 13 *KISS1* ^−/−^, 29 *KISS1* ^+/−^ and 40 *KISS1* ^+/+^; 44 males and 38 females. For second matings, five and one of the previously chosen F0 gilts and boars, respectively, were bred again. Eighty liveborn F1 piglets were farrowed through five litters (35 males and 30 females; average of 16 ± 1.7 piglets per litter), from which 21 piglets were *KISS1* ^−/−^, 31 *KISS1* ^+/−^ and 13 *KISS1* ^+/+^ (Table 2). In nine piglets farrowed in four litters (from 47FY and 65FY), only the paternal alleles were detected (Figures 1D, 5), suggesting that these two mothers harbored alleles with large deletions that could not be detected with the implemented PCR test. Inherited alleles of genotyped F1 piglets are shown in Figure 5 according to the litter.

**TABLE 2:** Summary of the generation of F1 piglets harboring *KISS1*-disrupted alleles.

Description		Round of matings		Altogether
		First	Second	
F0 pigs chosen to be bred	Boars	3	1	3
	Gilts/sows	10	7	10
Matings	Artificial insemination	7	5	12
	Natural	2	0	2
Pregnancy rate		78% (7/9)	100% (5/5)	86% (12/14)
Farrowed litters		7	5	12
Born piglets	All born	107	94	201
	Stillbirth rate	12% (13/107)	15% (14/94)	13% (27/201)
	Mummification rate	4% (4/107)	0% (0/94)	2% (4/201)
	Liveborn	90	80	170
	Genotyped^a^	82	65	147
	Alive at weaning	77	65	142
Liveborn piglets per litter	Mean ± SE	12.9 ± 1.6	16 ± 1.7	14.2 ± 1.2
	Maximum	18	20	20
	Minimum	7	11	7
Breed of genotyped piglets	Yorkshire	64	0	64
	Duroc	10	0	10
	Crossbred (Duroc × Yorkshire)	8	65	73
Sex of genotyped piglets	Male	44	35	79
	Female	38	30	68
Genotyped piglets	*KISS1* ^+/+^	40	13	53
	*KISS1* ^+/−^	29	31	60
	*KISS1* ^−/−^	13	21	34

^a^ Genotyping was focused on piglets that remained alive within the first days after birth. Post-birth mortality was mainly due to piglet crushing.

## 3 Discussion

In laboratory and livestock species, function of the kisspeptin system is needed for the proper regulation of gonadotropin secretion and reproduction (Seminara et al., 2003; d’Anglemont et al., 2007; Sonstegard et al., 2017). However, empirical evidence about the role of *KISS1* in pig reproduction is required to postulate that disruption of the gene encoding the kisspeptin ligand can be useful as an alternative to surgical castration. Here, we have generated four pigs harboring *KISS1* KO alleles in high frequencies that were phenocopies of humans and animals with HH. They were produced alongside *KISS1*-edited mosaic pigs that upon mating gave origin to 34 *KISS1* ^−/−^ F1 piglets, which confirmed the germline transmission of edited alleles. The generation of the *KISS1* KO F0 individuals shows that the kisspeptin ligand plays a determinant role in initiating the puberty of pigs. We observed that some animals with >80% *KISS1*-disruption still achieved sexual maturity, suggesting that even low concentrations of this peptide can trigger puberty.

Mosaicism in gene-edited zygotes is affected by variation in concentrations of gene editing reagents (Zhou et al., 2015; Tanihara et al., 2019; Menchaca et al., 2020). In this study, we sought to produce heterozygous-like KO founders to enable their breeding and generation of mosaic-free offspring for eventual deeper phenotyping. To accomplish this, we optimized sgRNA/Cas9 and ssODN levels *in vitro* (data not shown) and also co-injected two ssODNs, one designed to knock out *KISS1*, and the second designed to repair *KISS1* with silent alterations in the sgRNA biding site to prevent further cleavage. This combined approach resulted in intermediate editing with variable mosaicism in the F0 population, allowing puberty and breeding of subsequent generations. While mosaicism in this case was the intent, it is a common outcome in embryo editing and occurs when the genome editor persists past the one-cell stage. This results in unequal allele frequencies and/or more than two different alleles within the same individual (Mehravar et al., 2019; Hennig et al., 2020).

Although mosaicism can confound phenotyping in founder animals, when the latter is analyzed together with the genotypes, some clues about the function of the gene may be obtained. Four mosaic pigs with elevated *KISS1*-disruptive allele frequencies (>90%), displayed typical phenotypes of individuals with non-functional *KISS1*; hypogonadotropism, hypogonadism, failure to achieve puberty, and lack of gamete production (d’Anglemont et al., 2007; Lapatto et al., 2007; Uenoyama et al., 2015; Ikegami et al., 2020). In agreement with the heterozygosity of *KISS1* mutations (Lapatto et al., 2007), pigs of intermediate *KISS1*-disruptive editing (30%–70%) appeared to be reproductively normal. The absence of HH was also observed in pigs with above-intermediate frequency (>70%–90%) and even in a gilt (67FY) with considerably high frequency (97%) of *KISS1*-disruptive alleles. Further, ovulation was confirmed by postmortem evaluation of ovaries and serum concentrations of progesterone (data not shown) in some gilts of >80% *KISS1*-disruption. This suggests that a low amount of unaltered KISS1 is sufficient to stimulate the hypothalamic release of GnRH to support gonadotropin secretion for gonadal maturation and puberty, as evidenced by the pattern of LH secretion between highly edited individuals. Studies with rodents suggest that activation of only 10%–20% of GnRH neurons is necessary for an ovulatory surge of LH to be generated (Greig and Weisz, 1973; Gosden and Everett, 1976; Herbison et al., 2008). It is unknown how many kisspeptin neurons are required to activate this proportion of GnRH neurons, but with the data herein it can be hypothesized it is few.

In this regard, it is important to consider that *KISS1* allele frequencies clearly differed between tail tissue and sperm samples in three out of five mosaic F0 boars tested. If the same happened between hypothalamic and tail tissues, disparity could contribute to the absence of HH in some pigs classified as having high *KISS1*-disruptive mosaicism (>85%; e.g., 15MD and 67FY). This can occur when the tail tissue used for genotyping provides an allele frequency similar to the one observed in KO animals, but the hypothalamic region where KISS1 is produced has a lower *KISS1*-disruptive allele frequency to the point there are functional neurons to produce enough kisspeptin. Differences in allele frequencies among organs may explain why gilt 67FY was not KO with a *KISS1*-disruptive editing percent of 97% but that 49FY was KO with a *KISS1*-disruptive allele frequency of 96%. The discrepancy of *KISS1* allele frequencies between tail tissue and semen samples ranged between 2% and 43%. Similarly, Tanihara et al. (2020) reported that their greatest difference in allele frequency was 38% when comparing different organs from a single mosaic pig derived from editing embryos with CRISPR/Cas9. Another consideration is that WT cells in mosaic embryos could have preferentially developed into neurons by the process of blastocyst complementation (Kobayashi et al., 2010). However, that outcome is unlikely since *KISS1* KO does not prevent the formation of neurons, eliminating the selective pressure for WT neurons to out-compete *KISS1* KO neurons.

Overall, patterns of serum hormones during development for both gilts and boars were consistent with expected responses for pigs (Allrich et al., 1982; Berardinelli et al., 1984; Lutz et al., 1984; Ford et al., 2001; Barb et al., 2010) with the exception of FSH in boars (Schinckel et al., 1984), but this latter observation was not due to disruption of *KISS1* alleles. Taken together, there was no effect of gene editing on LH in gilts with >90% of *KISS1*-disruption, but FSH secretion early in development was suppressed in these animals. Serum concentrations of LH in boars with a frequency of *KISS1*-disruptive alleles >90% was severely suppressed throughout development. This in turn resulted in dramatic reduction in testosterone production and lack of age-related increases in circulating concentrations of testosterone expected for boars over a similar age range (Ford et al., 2001). Lower serum concentrations of testosterone in boars with high levels of *KISS1* disruption portends that they would likely have a reduction in other androgen compounds (androstenone and skatole) that would cause boar taint (Zamaratskaia and Squires, 2009).

Phenotypical variability for hypogonadism, uterine weight, and other similar reproductive-related observations have been described in *Kiss1* KO and *Kiss1r* KO rodents (d’Anglemont et al., 2007; Lapatto et al., 2007; Uenoyama et al., 2015). We are currently assessing these phenotypes in *KISS1*-disrupted F1 pigs lacking mosaicism. Although bilateral cryptorchidism was reported in a human and rats with impaired kisspeptin systems (Semple et al., 2005; Uenoyama et al., 2015), there is no clear link between these conditions. In this study, one pig presented unilateral cryptorchidism, but its frequency of *KISS1*-disruptive alleles was considerably lower (60%) than that of *KISS1* KO boars (100%). Cryptorchidism is one of the most common congenital defects in pigs (Mattsson, 2011) and it that impaired testicular descent of boar 53MY is likely unrelated to *KISS1* disruption.

Spermatozoa can typically be found in histological sections of pig testes by 120 days (Malmgren et al., 1996; Lee et al., 2014). Testicular histology of *KISS1* KO pigs, at 255–262 days of age, showed a lack of spermatozoa, with atrophied seminiferous tubules, but otherwise normal in structure. Similar findings in *KISS1R* KO pigs were reported by Sonstegard et al. (2017), who observed hypogonadism and lack of gamete production in KO boars. At 8 months of age, spermatogenesis was observed in the seminiferous tubules of WT boars, while in *KISS1* KO boars the germ cells were still randomly arranged within the tubules and failed to undergo spermatogenesis. This distribution pattern of germ cells is typical of pigs whose ages range between five and 60 days. Boar germ cells have been reported to complete migration to the basement membrane of the seminiferous tubule by 90 days of age (Lee et al., 2014). It is believed that the placement of germ cells at the basement membrane is an important environmental stimulus for further maturation (Nagano et al., 2000). Impaired germ cell relocation may be one of the events preventing spermatogenesis in *KISS1* KO boars.

Breeding of mosaic F0 boars and gilts resulted in non-mosaic F1 piglets, some of them harboring *KISS1*-disruptive alleles in either the heterozygous or homozygous state. Two F0 mothers transmitted alleles to some of their piglets that were not amplified due to the deletion of at least one of the primer-annealing sites, which are required for genotyping of the targeted locus. This indicates the presence of on-target deletions larger than detected by our PCR assay. The occurrence of these large deletion events at a target site has been documented using CRISPR/Cas9-mediated editing of embryos in different species (Owens et al., 2019; Koppes et al., 2020; Korablev et al., 2020; Alanis-Lobato et al., 2021; Höijer et al., 2022). A number of aspects could affect the presence and abundance of larger indels at on-target sites such as the delivery method of the CRISPR/Cas9 system, its concentration, and the use of HDR donor templates (Wen et al., 2021; Höijer et al., 2022). It is thought that the differential kinetics of non-homologous end joining, HDR, or microhomology-mediated end joining repair machinery influence the frequency of large deletions (Wen and Zhang, 2022).

Generation of offspring from F0 pigs confirmed allele transmission. Mosaic founders with *KISS1* disruption between 25% and 70% had normal pregnancy rates and litter sizes in the first and second parity. KISS1 is attributed a role in pregnancy as this peptide is believed to inhibit trophoblast migration and invasion in the placenta (Cao et al., 2019). In the case of boars, Zou et al. (2019) reported a positive relationship between KISS1 concentration in seminal plasma and total sperm count as well as total motile sperm count. Although minimal numbers of ejaculates were assessed in the current study, they were similar to what one would expect for commercial boars. Volume and quality of ejaculate increased as boars became fully trained to semen collection.

Variation in growth-related traits was not affected by the percent of *KISS1* disruption (Figures 2G–I), and F0 *KISS1* KO pigs were within the average for the swine industry. Similar results were found by Lapatto et al. (2007) but not by d'Anglemont et al. (2007) for the body weight of *Kiss1* KO mice compared to WT mice. Likewise, *Kiss1* KO rats were reported to have normal growth (Uenoyama et al., 2015). No growth impairment due to *KISS1* disruption in swine was expected because, unlike in sheep and cattle (Kadokawa et al., 2008; Whitlock et al., 2008; Foradori et al., 2017), there is no link between kisspeptin and the somatotropic axis in pigs (Lents et al., 2008). This is relevant because adequate growth performance of KISS1-deficient pigs is necessary for wide acceptance in the swine industry.

Demonstrating that *KISS1* KO in pigs prevents puberty supports conducting additional performance studies at the appropriate statistical power. This will enable comparison not only of growth, but also of other phenotypes of interest like feed efficiency and boar taint compound levels from KO and WT animals. While these studies are essential for producer and consumer acceptance, they are not warranted until a breeding strategy can be established. The results in this manuscript provide valuable histological and hormonal results for use as a baseline in follow-on fertility restoration studies.

Producers are not likely to adopt approaches in which perpetuating the trait involves gene editing each litter or insertion of foreign DNA sequences into the genome of the breeding stock. For example, knocking out the SRY gene to induce male-to-female sex reversal would require direct editing of embryos for every litter, and integration into the parental genomes of a CRISPR/Cas vector would be needed for the production of only-female litters (Kurtz et al., 2021). Likewise, editing genes to reduce the biosynthesis of boar taint precursors would be difficult to implement due to the large number of enzymes involved, and steroid biosynthetic precursors can flow to synthetic endpoints through multiple pathways (Rydhmer et al., 2006; Squires et al., 2019). Alternatives in which both boar taint and aggressiveness can be prevented and spread through selective breeding are more likely to be embraced by producers.

The current results demonstrate that kisspeptin ligand activation of the hypothalamic-pituitary-gonadal axis is required to induce puberty in pigs. Other mechanisms in this species cannot autonomously compensate for the loss of *KISS1* function. These findings also confirm the inheritance of *KISS1*-disruptive alleles and suggest that a low amount of functional KISS1 may be required to trigger the onset of puberty in pigs. The goal of this and ongoing research with the F1 generation is to improve the welfare of millions of pigs globally, and the safety of the personnel who handle them, by preventing surgical castration of male piglets, while ensuring good pork quality. Achieving this will depend on further investigation to provide critical information about how to manage the reproductive phenotypes of these *KISS1*-edited animals.

## 4 Materials and methods

### 4.1 Ethics approval

All animal procedures followed institutional guidelines for the care and use of animals and were approved by the Recombinetics Institutional Animal Care and Use Committee (IACUC; approval number: RCI-1903-03A-A3).

### 4.2 Animals

The F0 pigs were of Duroc or American Yorkshire breeding, derived from commercial herds, and farrowed from first and second parity surrogate sows. The F0 boars and gilts chosen for breeding resulted in both purebred and crossbred animals. An industry standard fortified corn-soybean meal diet formulated to meet and exceed nutrient requirements was provided with *ad libitum* access to water.

### 4.3 Generation of *KISS1*-edited pigs using sgRNA/Cas9 RNPs and ssODNs

*Sus scrofa KISS1* possesses a genomic structure of two exons that encode 138 aa. Amino acids 111–120 form a 10 aa core conserved across vertebrates that is essential for binding and activation of KISS1R. A sgRNA was designed to target a *KISS1* exonic region preceding the sequence encoding this completely conserved aa core motif. The online tools Gene Sculpt Suite MENTHU (Ata et al., 2018) and microhomology predictor (Bae et al., 2014) were used to design the sgRNA with the sequence of 5′-GGT​CCC​CCG​AGG​GTT​CGC​CTCGG-3′ (PAM is underlined). crRNA and tracrRNA (IDT, IA, United States) were resuspended in injection buffer (5 mM Tris-HCl, 0.1 mM EDTA, pH 7.5) and incubated with HiFi Cas9 protein (IDT, IA, United States) to form RNP complexes. The assembled RNPs were co-injected in fertilized porcine zygotes with two ssODNs of 90 bp (IDT, IA, United States) that served as templates for HDR-mediated editing (Figure 1B). The first ssODN, HDR^SC^, introduces an 8-bp insertion to generate a premature termination codon followed by the HindIII restriction site. The second ssODN, HDR^BM^, produces two silent mutations at and close to the PAM site that reduce Cas9 recutting after HDR^BM^ editing while also introducing the restriction site AcuI.

Single-cell embryos for microinjection were produced *in vivo* by synchronizing the ovulation of 71 sows with 200 μg of the GnRH agonist triptorelin acetate (OvuGel; JBS United Animal Health, IN, United States) 96 h (h) post-weaning. At 22 ± 2 h after triptorelin acetate treatment, sows were artificially inseminated with extended boar semen. The following day, sows were transferred to the slaughterhouse at the Meat Science Laboratory at the University of Minnesota, where the reproductive tracts were collected. The zygotes were recovered by flushing the oviduct with phosphate-buffered saline (1X PBS; Corning, NY, United States). A total of 836 *in vivo* fertilized zygotes were obtained. Single-cell embryos were immediately transferred into TCM-199 medium (Gibco, MD, United States) and 3–5 h after zygote recovery, 684 one-cell stage embryos were injected. Zygote microinjection was done into the cytoplasm with sgRNA (25 ng/μl)/Cas9 (50 ng/μl) RNPs and ssODNs (HDR^SC^: 33.3 ng/μl; HDR^BM^: 66.7 ng/μl) using an inverted microscope (Nikon, Tokyo, Japan), which was equipped with micromanipulators (Narishige, Tokyo, Japan) and an electronic microinjection system (Femtojet; Eppendorf, Hamburg, Germany). Injected zygotes were cultured for 12–16 h in PZM-3 medium (Yoshioka et al., 2002) covered with mineral oil, at 38°C in a humidity-controlled atmosphere containing 5% CO_2_ and 5% O_2_.

Within approximately 20–24 h from zygote collection, an average of 36 microinjected zygotes were surgically transferred into oviducts of 19 hormonally synchronized surrogate sows. To synchronize the estrous of recipient sows, they were treated with 18 mg/day of altrenogest (MATRIX; Merck & Co., NJ, United States) for 18 days, plus 1,000 IU of human chorionic gonadotropin (hCG; Chorulon, Merck & Co., NJ, United States) on day 19, and embryo transfers were carried out 48 h later. Pregnancies were confirmed and monitored on days 30 and 60 after the embryo transfers using an Aloka 500 Ultrasound Scanner (Aloka Co. Ltd., CT, United States). After 115–118 days of gestation, 10 recipient females farrowed naturally, and piglets were genotyped.

### 4.4 Genotyping

#### 4.4.1 F0 and F1 pigs

Genomic DNA was extracted from tail docking biopsies of all (alive or dead) F0 and alive F1 piglets born using the DNeasy Blood and Tissue Kit (QIAGEN, MD, United States). DNA was subjected to PCR amplification of the CRISPR/Cas9 targeted locus with specific primers (F: 5′-GGATGAGCAAACGGTCCAGA-3′and R: 5′-CTC​CCG​GGT​TTG​AAG​GTC​TC-3′; WT amplicon size: 409 bp) using AccuStart II GelTrack PCR SuperMix (QuantaBio, MA, United States). Amplicons were purified with the QIAquick PCR Purification Kit (QIAGEN, MD, United States) and sent to Sanger sequencing (ACGT Inc., IL, United States) for both F0 and F1 pigs. The resulting trace files were analyzed with the bioinformatics package ICE (Conant et al., 2022). For a better quantification of the mutations and more accurate deciphering of genotype in pigs generated by embryo editing, amplicons of 45 F0 pigs were sequenced using Amplicon-EZ NGS (GENEWIZ, NJ, United States). These 45 piglets were chosen because the Sanger sequencing results indicated that they could have the potential to be used as parents to generate the *KISS1*-edited F1 generation, or that they could be *KISS1* KO animals.

NGS data was demultiplexed with Geneious 7.1.9^1^. Reads were analyzed using the CRISPR RGEN Tools Cas-Analyzer software (Park et al., 2017), which provided a detailed breakdown of the allele frequency for the targeted *KISS1* locus. Predicted translations of the detected alleles, as well as DNA and protein sequence alignments, were generated utilizing Geneious 7.1.9; this information was used to estimate the potential of the generated alleles to disrupt *KISS1*. Two alleles were classified as of unknown translation since their corresponding Sanger trace files did not allow proper allele deciphering (Figure 1D).

#### 4.4.2 Semen of *KISS1*-edited boars

Ejaculated semen samples from F0 boars were subjected to genomic DNA isolation using the QIAamp DNA Mini Kit and an adapted QIAamp Tissue Protocol (QIAGEN, MD, United States). DNA obtained from semen was PCR-amplified, amplicons were purified as described above, and PCR products were NGS-sequenced. Amplicons were submitted for NGS to the US Meat Animal Research Center (NE, United States) and downstream analyzed as described in the previous paragraph.

### 4.5 Phenotypic measures

Body weights of all F0 pigs were individually recorded within 48 h after birth (birth weight) and at 40, 70, 100, 130, and 160 days of age ±4 days. The growth rate was calculated as the average daily gain of weight for the time periods birth-40, 40–70, 70–100, 100–130, and 130–160 days of age. The total average daily gain of weight was estimated from birth to 160 days of age. Beginning at 40 days of age and continuing, at each time body weight was collected, the length and width of both testicles were individually measured with a vernier caliper (35-OD8; iGaging, CA, United States).

Testicular volume was estimated using the equation for a prolate spheroidal shape (Young et al., 1986): $TV=4/3\cdot \pi \cdot a\cdot b^{2}\cdot 2$ , where TV is testis volume (cm^3^), π is 3.14, a is ½ length testis (cm), and b is ½ width testis (cm). This equation was calculated by measuring the width and length of both testes, besides subtracting the skinfold thickness (Sinclair et al., 2001; Jacyno et al., 2015; Han et al., 2017). A Harpenden skinfold caliper was employed for measuring the skinfold thickness (2 layers of scrotal skin) (Jacyno et al., 2015).

Blood samples were collected from F0 pigs monthly starting at 40 days of age and biweekly from 130 to 280 days of age by jugular venipuncture into serum-separating tubes. Serum was obtained by centrifugation at 1,500 × *g* for 15 min (min) at 4°C, aliquoted, and stored at −80°C until analysis. Serum concentrations of LH (Kesner et al., 1987), FSH (Trout et al., 1992), testosterone (Ford et al., 2001), and progesterone (Calderón et al., 2017) were quantified with validated radioimmunoassay. Testosterone and progesterone assays were obtained from MP Biomedicals (Irvine, CA, United States). The reference standards for LH (AFP-10506A) and FSH (AFP-10640B) were provided by Dr. A. F. Parlow (Scientific Director of the NIH, NIDDK, National Hormone and Peptide Program, Torrance, CA, United States). Pools of porcine serum were included in each assay. For LH, concentrations of pools measured .81, 1.54, and 8.41 ng/ml with an average intra- and inter-assay coefficient of variation (CV) of 6.7% and 10.8%, respectively (*n* = 8 assays). For FSH, serum concentrations of pools ranged from 2.0 to 6.8 ng/ml with average intra- and inter-assay CV of 6.3% and 7.3%, respectively (*n* = 6 assays). For progesterone, a serum pool (midluteal phase) measuring 24.2 ng/ml had an intra- and inter-assay CV of 5.7% and 8.6%, respectively (*n* = 4 assays). Intra- and inter-assay CV were 10.7% and 13.8% (*n* = 6 assays), respectively, for a pool of boar serum with testosterone concentration of 5.3 pg/ml.

### 4.6 Gross morphology

Pigs were humanely euthanized according to established guidelines (American Veterinary Medical Association, 2020) for tissue collection. The length and width of ovaries and testes were individually measured with a vernier caliper, and the number of ovarian follicles was counted. Ovaries and testes were individually weighed. External and internal genitalia were observed for visible abnormalities. The number of pigs that were evaluated postmortem as well as their minimum and maximum ages at this point, for each *KISS1*-disruption group, are presented in Supplementary Tables S2, S3.

### 4.7 Histology and immunohistochemistry

Testes from F0 boars at 255–262 days of age were collected, bivalved, cut through the longest dimension, and 0.5 mm × 0.5 mm pieces containing sections of mediastinum and periphery were collected. These tissues were placed into 15 ml conical tubes with 10% Neutral Buffered Formalin (89370-94; VWR, PA, United States), and maintained at room temperature for 18 h. Tissues were then washed three times in PBS with rocking for 10 min. Samples were placed in 70% ethanol (V1001TP; Decon Laboratories Inc., PA, United States) and stored at room temperature until submission to Scientific Solutions (MN, United States) for paraffin embedding. Tissue sections of 20 microns were mounted on slides (Globe Scientific Inc., NJ, United States) and deparaffinized with Xylene (89370-008; VWR, PA, United States) followed by rehydration with increasing concentrations of 70%–100% ethanol. Antigens were retrieved by boiling the tissue in a microwave for 15 min with a citrate-based unmasking solution (1:100; H-3300; Vector Laboratories Inc., CA, United States). Membrane permeabilization was accomplished by incubating tissue sections in PBS containing 0.1% Triton X-100 (Sigma-Aldrich, MO, United States) for 10 min at room temperature. To block the binding of non-specific proteins, tissue sections were incubated with 10% normal goat serum (Sigma-Aldrich, MO, United States) in PBS containing 0.025% tween-20 (PBST; Bio-Rad, CA, United States) overnight at 4°C. Washes with PBS were done twice for 10 min after blocking, primary antibody stain, secondary stain, and DAPI staining.

Tissue sections were incubated overnight at 4°C with primary antibodies diluted to manufacturer recommendations with 5% normal goat serum-PBST. Vimentin (1:500 dilution; Abcam, Cambridge, UK) and GATA binding protein 4 (GATA4; 1:500 dilution; Cell Signaling Technology, MA, United States) were used as markers for Sertoli cells. Deleted in azoospermia-like protein (DAZL; 1:500 dilution; Abcam, Cambridge, UK) and protein gene product 9.5 (PGP9.5; 1:1,000 dilution; Abcam, Cambridge, UK) were used as germ cells markers. Tissue sections were incubated for 1 h at room temperature in secondary antibodies diluted 1:400 in 5% normal goat serum-PBST. These antibodies were Alexa Fluor 488 (anti-rabbit) or Alexa Fluor 594 (anti-mouse; Invitrogen, MA, United States). Details of antibodies used are in Supplementary Table S6. Nuclei were stained with Hoechst solution (Thermo Fisher Scientific Inc., MA, United States) diluted 1:10,000 in PBS for 15 min. Tissue sections were washed with PBS (5 min, room temperature) and coverslipped using ImmuMount (Fisher Scientific, MA, United States), followed by incubation at room temperature for 30–120 min. A Leica DM6000B microscope equipped with a Leica DFC7000 T camera (Leica Microsystems, Wetzlar, Germany) was used to view and image slides.

### 4.8 Statistical analysis

Descriptive statistics of all anatomical-related phenotypes and statistical analyses of body weight, average daily gain, and hormone concentration were estimated using SAS 9.4 (SAS Institute Inc., NC, United States). The WT group always consisted of all pigs that harbored 0% of *KISS1*-disruptive alleles, including individuals carrying *KISS1*-non-disruptive alleles at different frequencies as these did not differ phenotypically from unedited pigs.

#### 4.8.1 Body weight and growth rate

The statistical model for birth weight (*n* = 58) included fixed effects of sex, *KISS1*-disruption group (WT, 5%–90%, or >90%), and their interaction along with a random litter effect (nine levels). Body weight and average daily gain (*n* = 287 each) were analyzed as repeated measures with fixed effects of sex, *KISS1*-disruption group, age (five levels), and their two- and three-way interactions. Birth weight was included as a linear covariate. A type I autoregressive (co)variance structure with heterogenous variance across age was chosen to model the random pig effect.

#### 4.8.2 Hormone analysis

Box plots were initially constructed using all available observations of boar testosterone (*n* = 234), boar LH (*n* = 233), boar FSH (*n* = 235), gilt LH (*n* = 246), and gilt FSH (*n* = 247) and outliers were identified as those having values greater or less than three times the interquartile range within each sampling age. After removing outliers, 226, 231, 240, and 244 observations remained for analysis of boar testosterone, boar LH, gilt LH, and gilt FSH, respectively. Little variability was observed in boar FSH after 160 days of age and these data were removed, leaving 169 observations for analysis. A natural logarithm transformation was applied to all hormones, which were analyzed as repeated measures within sex with fixed effects of *KISS1*-disruption group (WT, 5%–90%, or >90%), age (up to nine levels), and their interaction. A type I autoregressive (co)variance structure with heterogenous variance across age was chosen to model the random pig effect. Least-squares means and their standard errors were back-transformed to the scale of measurement using the delta method.

### 4.9 Semen collection of *KISS1*-edited boars

F0 boars that were candidates to be bred were trained on a semen-collection dummy. When mosaic boars were between 272 and 329 days of age, semen was collected *via* the hand-gloved method. Ejaculates were quantified, samples were sent for NGS-mediated genotyping as outlined above, and replicate semen was extended for evaluation of sperm motility, morphology, and concentration with a light microscope (CxL; Labomed, CA, United States).

### 4.10 Artificial insemination using *KISS1*-edited boars and gilts

In mosaic gilts that were candidates to be mated due to their genotypes, estrus was synchronized using altrenogest for at least 14 days. Gilts that displayed classical signs of estrus were artificially inseminated with semen of F0 boars bearing *KISS1*-disruptive alleles. First matings by artificial insemination occurred when gilts were between 275 and 355 days of age and boars were 317 and 348 days old. A second mating was performed 9 months later. Semen collections and pregnancy diagnoses were done as previously mentioned.

## Data Availability

The original contributions presented in the study are included in the article/Supplementary Materials, further inquiries can be directed to the corresponding author.

## Footnotes

1
https://www.geneious.com.

## Author Disclaimer

Mention of trade names or commercial products in this publication is solely for the purpose of providing specific information and does not imply recommendation or endorsement by the USDA. The USDA prohibits discrimination in all its programs and activities on the basis of race, color, national origin, age, disability, and where applicable, sex, marital status, familial status, parental status, religion, sexual orientation, genetic information, political beliefs, reprisal, or because all or part of an individual’s income is derived from any public assistance program (not all prohibited bases apply to all programs). Persons with disabilities who require alternative means for communication of program information (Braille, large print, audiotape, etc.,) should contact USDA’s TARGET Center at (202) 720-2600 (voice and TDD). To file a complaint of discrimination, write to USDA, Director, Office of Civil Rights, 1400 Independence Avenue, S.W., Washington, DC 20250-9410, or call (800) 795-3272 (voice) or (202) 720-6382( TDD). USDA is an equal opportunity provider and employer.
