# Supplementary material for: CRISPR/Cas9-editing of KISS1 to generate pigs with hypogonadotropic hypogonadism as a castration free trait
Source: Front Genet. 2023 Jan 4;13:1078991. doi: 10.3389/fgene.2022.1078991 (PMC9854396; doi:10.3389/fgene.2022.1078991)
Supplement: Supplementary file 1 [file DataSheet1.pdf]

Supplementary Material

Supplementary Table S1 | Generated pigs after targeting *KISS1* via microinjection of CRISPR/Cas9 RNPs and ssODNs into *in vivo* derived zygotes.

Description	Number
Donor sows	71
Collected zygotes	836
Injected and transferred zygotes	684
Recipient sows	19
Average of transferred embryos/recipient	36
Recipient pregnancy rate	53% (10/19)
Litters of liveborn piglets	9
Survival rate of microinjected zygotes*	21%
Efficiency rate of microinjected zygotes**	10%
Total of born piglets	74
Liveborn piglets	69
American Yorkshire males	27
American Yorkshire females	22
Duroc males	8
Duroc females	12
Stillbirth rate of liveborn litters	7% (5/74)
Post-birth mortality rate (piglet crushing)	12% (8/69)
Excluded piglets due to common medical conditions	3

*Average of piglets born alive per embryos transferred to a pregnant recipient. **Average of piglets born per total embryos transferred.

Supplementary Table S2 | Descriptive statistics of testicular volume and weight in gene-edited and WT boars according to their *KISS1*-disruptive editing percent.

Phenotype	*KISS1*-disruptive editing percent	Days† or description	Mean (SD)	Minimum	Maximum	*n*
Testicular volume throughout development (cm³)	>90%	40	1.4 (1.6)	0	2.3	2
		70	2.1 (2.9)	0	4.1	2
		100	1.5 (2.1)	0	3	2
		130	0	0	0	2
		160	0	0	0	2
		190	0	0	0	2
	5-90%	40	14.9 (7)	1.4	25.7	12
		70	24.4 (12.3)	2.9	41.2	14
		100	65.1 (37.6)	14	136.6	13
		130	249.4 (175.1)	45.6	637	14
		160	566 (244)	62	1088	14
		190	748 (244.8)	229	1102	11
	WT	40	12.1 (4.9)	1.5	19.1	11
		70	23.5 (7.8)	10.9	37.3	13
		100	63.4 (36.3)	22.3	126.4	13
		130	188.8 (91.1)	110.9	385.4	13
		160	471.6 (159.1)	263.6	791.8	11
		190	830 (191.3)	575.9	1167	10
Ex vivo testicular volume (TV; cm³)	>90%	TV	5.8 (1.9)	4.4	7.1	2
		Age*	244.5 (24.8)	227	262	2
	5-90%	TV	914.6 (235.6)	748.8	1369	6
		Age*	255.3 (50.1)	211	337	6
	WT	TV	678.2 (255.3)	214.7	990.8	10
		Age*	217.5 (44.1)	158	262	10
Testicular weight (g)	>90%	Right testicle	17 (18.4)	4	30	2
		Left testicle	16.5 (19.1)	3	30	2
		Age*	244.5 (24.8)	227	262	2
	5-90%	Right testicle	460.3 (121)	305	621	6
		Left testicle	474.7 (90.8)	335	578	6
		Age*	255.3 (50.1)	211	337	6
	WT	Right testicle	344.3 (108.6)	119	451	10
		Left testicle	346.7 (104.3)	144	449	10
		Age*	217.5 (44.1)	158	262	10

SD, standard deviation; †± 4 days except when it is day 1, and day 1 ranges between 0 and 48 h; *days.

Supplementary Table S3 | Descriptive statistics of follicle count, ovary measurements, and ovary weight in gene-edited and WT gilts according to their *KISS1*-disruptive editing percent.

**Phenotype**	***KISS1*-disruptive editing percent**	**Description**	**Mean (SD)**	**Minimum**	**Maximum**	***n***
Follicle number	>90%	Right ovary	16 (27.7)	0	48	3
		Left ovary	18 (31.2)	0	54	3
		Age*	243.7 (25.9)	214	262	3
	5-90%	Right ovary	23.9 (16.2)	10	50	7
		Left ovary	25.9 (18.2)	10	61	7
		Age*	289.7 (53.6)	222	374	7
	WT	Right ovary	44 (16.3)	21	70	8
		Left ovary	38.5 (13.7)	20	57	8
		Age*	255.4 (26.7)	214	288	8
Ovary measurements (cm)	>90%	Right ovary length	2.3 (2)	1	4.6	3
		Left ovary length	2.1 (1.7)	1.1	4.1	3
		Right ovary width	1.3 (1.1)	0.5	2.6	3
		Left ovary width	1.4 (1.3)	0.5	2.8	3
		Age*	243.7 (25.9)	214	262	3
	5-90%	Right ovary length	3.5 (1)	2.3	5.2	7
		Left ovary length	3.8 (1.4)	2.2	6.5	7
		Right ovary width	2 (0.4)	1.5	2.5	7
		Left ovary width	2.3 (1)	1.3	4.3	7
		Age*	289.7 (53.6)	222	374	7
	WT	Right ovary length	3.4 (0.8)	2.2	4.5	8
		Left ovary length	3.4 (0.9)	2.1	4.5	8
		Right ovary width	2.6 (0.5)	2	3.5	8
		Left ovary width	2.4 (0.6)	1.5	3	8
		Age*	255.4 (26.7)	214	288	8
Ovary weight (g)	>90%	Right ovary	5.8 (9.7)	<1	17	3
		Left ovary	3.1 (5.1)	<1	9	3
		Age*	243.7 (25.9)	214	262	3
	5-90%	Right ovary	7.6 (4.3)	4	16	7
		Left ovary	13 (12.2)	6	40	7
		Age*	289.7 (53.6)	222	374	7
	WT	Right ovary	10.3 (4)	3	14	8
		Left ovary	10.6 (5.9)	2	18	8
		Age*	255.4 (26.7)	214	288	8

SD, standard deviation; *days.

Supplementary Table S4 | Descriptive statistics of body weight and growth rate in gene-edited and WT pigs according to their *KISS1*-disruptive editing percent.

Phenotype	***KISS1*-disruptive editing percent**	Days†	Mean (SD)	Minimum	Maximum	*n*
Body weight (kg)	>90%	1	1.7 (0.2)	1.4	1.9	5
		40	8.3 (1.4)	6.8	15.3	5
		70	27.6 (3.4)	22.2	31.8	5
		100	51.7 (3)	48.1	56.4	5
		130	80.3 (2.4)	76.8	83.3	5
		160	112.7 (8.2)	104.5	123.6	5
	5-90%	1	1.7 (0.3)	1.2	2.2	29
		40	11 (2.2)	6.8	16.3	29
		70	31.5 (4.5)	24.5	39.9	29
		100	57.3 (8.1)	43.3	76.2	29
		130	85.2 (11.5)	61.9	114.8	29
		160	118.9 (14.2)	85.4	156.4	29
	WT	1	1.5 (0.3)	0.8	2	24
		40	9.5 (2)	5	12.8	24
		70	28 (5)	16.3	34.5	24
		100	51.8 (8.5)	35.4	62.8	24
		130	79.1 (11.3)	50.8	91.7	24
		160	109.1 (12.1)	79.6	127.3	21
Average daily gain of weight (kg)	>90%	1-40	0.2 (0.03)	0.1	0.2	5
		40-70	0.6 (0.1)	0.5	0.8	5
		70-100	0.8 (0.2)	0.6	1	5
		100-130	1 (0.1)	1	1.1	5
		130-160	1.2 (0.3)	0.8	1.7	5
		Total (1-160)	0.7 (0.1)	0.7	0.8	5
	5-90%	1-40	0.2 (0.04)	0.2	0.4	29
		40-70	0.7 (0.1)	0.5	0.8	29
		70-100	0.9 (0.2)	0.5	1.3	29
		100-130	1 (0.1)	0.7	1.4	29
		130-160	1.1 (0.3)	0.7	2	29
		Total (1-160)	0.7 (0.1)	0.5	1	29
	WT	1-40	0.2 (0.04)	0.1	0.3	24
		40-70	0.6 (0.1)	0.3	0.8	24
		70-100	0.8 (0.1)	0.6	1	24
		100-130	1 (0.2)	0.4	1.2	24
		130-160	1 (0.2)	0.7	1.6	21
		Total (1-160)	0.7 (0.1)	0.5	0.8	21

SD, standard deviation; †±4 days except when it is day 1, and day 1 ranges between 0 and 48 h.

Supplementary Table S5 | Basic characteristics of ejaculates from mosaic *KISS1*-edited boars chosen as potential fathers of an F1 generation.

Boar	*KISS1*-disruptive editing percent (%)		Breed	Descriptive statistics	Ejaculate volume (ml)	Progressive motility (%)	Sperm morphology				Sperm concentration ($\times 10^6$/ml)
	Tail	Sperm					Intact (%)	Abnormalities (%) Tail	Midpiece	Head	
13MY	35	35	American Yorkshire	Mean ± SE	118.3 ± 70	70 ± 20	93.3 ± 1.7	1.7 ± 1.7	5 ± 0.6	0	
				Max.	290	90	96	5	6	0	1020
				Min.	3	30	90	0	4	0	
				CV (%)	118.4	49.5	3.3	173.2	20	-	
				Number of ejaculates	4	3	3				1
36MY	45	75	American Yorkshire	Mean ± SE	274.4 ± 55.1	95 ± 1.3	95.9 ± 1.4	2 ± 1.3	2.1 ± 1	0	
				Max.	475	100	100	10	8	0	750
				Min.	5	90	90	0	0	0	
				CV (%)	63.6	4	4.1	177.3	129.3	-	
				Number of ejaculates	10	8	8				1
53MY	60	38	American Yorkshire	Mean ± SE	207.5 ± 72	93.8 ± 2.4	92.3 ± 1.3	1.3 ± 0.5	6.3 ± 1	0	
				Max.	350	100	96	2	8	0	660
				Min.	15	90	90	0	4	0	
				CV (%)	69.4	5.1	2.9	76.6	33	-	
				Number of ejaculates	4	4	4				1
15MD	88	66	Duroc	Mean ± SE	65.3 ± 28.7	69.2 ± 15.4	83 ± 6.6	3.6 ± 0.7	13.4 ± 6.8	0	240

				Max.	190	95	96	5	30	0	
				Min.	2	0	66	2	0	0	
				CV (%)	107.6	54.6	17.8	42.1	113.9	-	
				Number of ejaculates	6	6	5				1
61MY	59	57	American Yorkshire	Mean ± SE	3	20	94	2	4	0	
				Max.	-	-	-	-	-	-	-
				Min.	-	-	-	-	-	-	
				CV (%)	-	-	-	-	-	-	
				Number of ejaculates	1	1	1				1

Supplementary Table S6 | Details of antibodies used.

Antibody name	Vendor	Catalog number	Clonality	Host organism	RRID
**Vimentin**	Abcam	Ab8069	Monoclonal	Mouse	AB_306239
**GATA4** (GATA binding protein 4)	Cell Signaling Technology	36966S	Monoclonal	Rabbit	AB_2799108
**DAZL** (Deleted in azoospermia-like protein)	Abcam	Ab215718	Monoclonal	Rabbit	AB_2893177
**PGP9.5** (Protein gene product 9.5)	Abcam	Ab8189	Monoclonal	Mouse	AB_306343
**Alexa Fluor 488**	Thermo Fisher Scientific	A-11070	Polyclonal	Goat	AB_2534114
**Alexa Fluor 594**	Thermo Fisher Scientific	A-11020	Polyclonal	Goat	AB_2534087

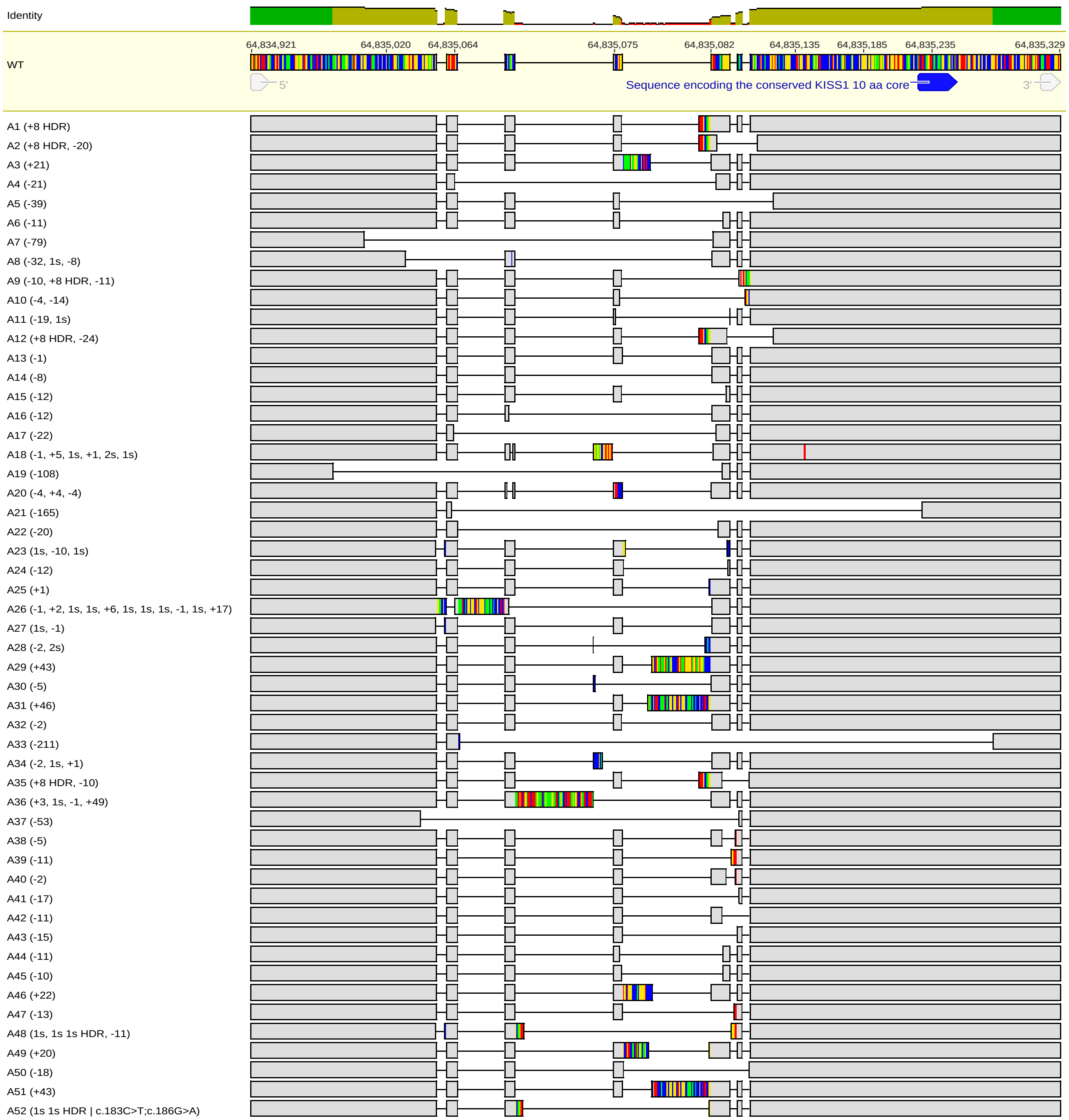

Supplementary Figure S1 | Nucleotide sequences of the detected *KISS1*-edited alleles aligned to the WT sequence of the *KISS1* amplified locus. Names of the detected alleles are on the left side and within parentheses are their corresponding edits. Differences between the WT and edited alleles are highlighted in these lasts. Alleles A38-44 were deciphered through only Sanger sequencing, and all other alleles were also decoded using high-throughput sequencing. “+” indicates insertion, “-” deletion, and “s” substitution of nucleotides. +8 homology-directed repair (HDR) and 1s 1s HDR refer to HDR$^{\text{stop codon}}$ and HDR$^{\text{blocking mutations}}$ edits, respectively.

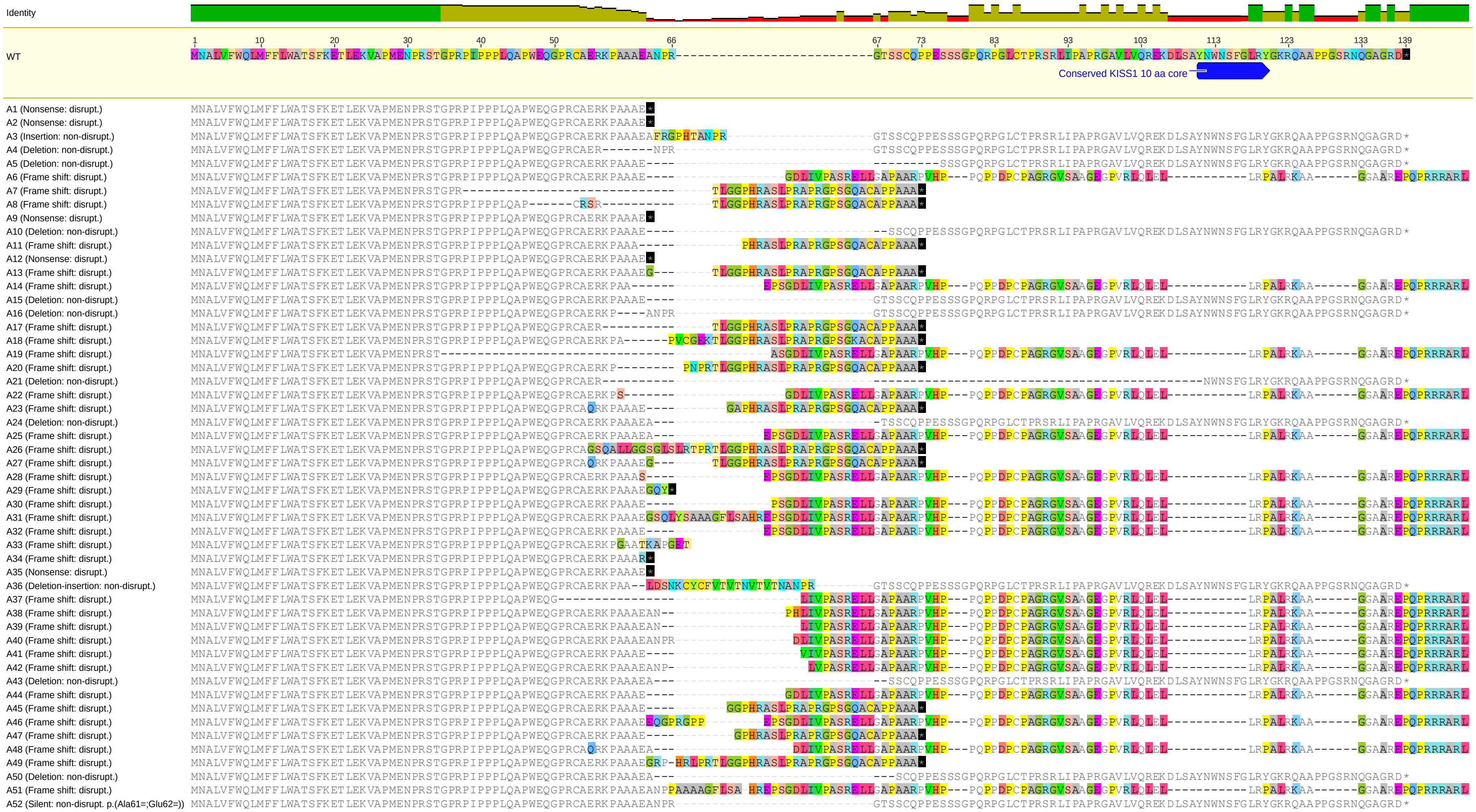

Supplementary Figure S2 | Predicted protein translation of the detected alleles and their alignment against the WT sequence of kisspeptin. The names of the detected alleles are on the left side. Within parentheses, the protein mutation type for each allele is written alongside their potential to disrupt KISS1 by altering its conserved 10 amino acid (aa) core. “Disrupt.” stands for disruptive whereas “non-disrupt.” for non-disruptive, and both refer to the predicted capacity of the allele to impair the function of kisspeptin. Differences between the protein sequences of the WT and edited alleles are highlighted in these lasts.

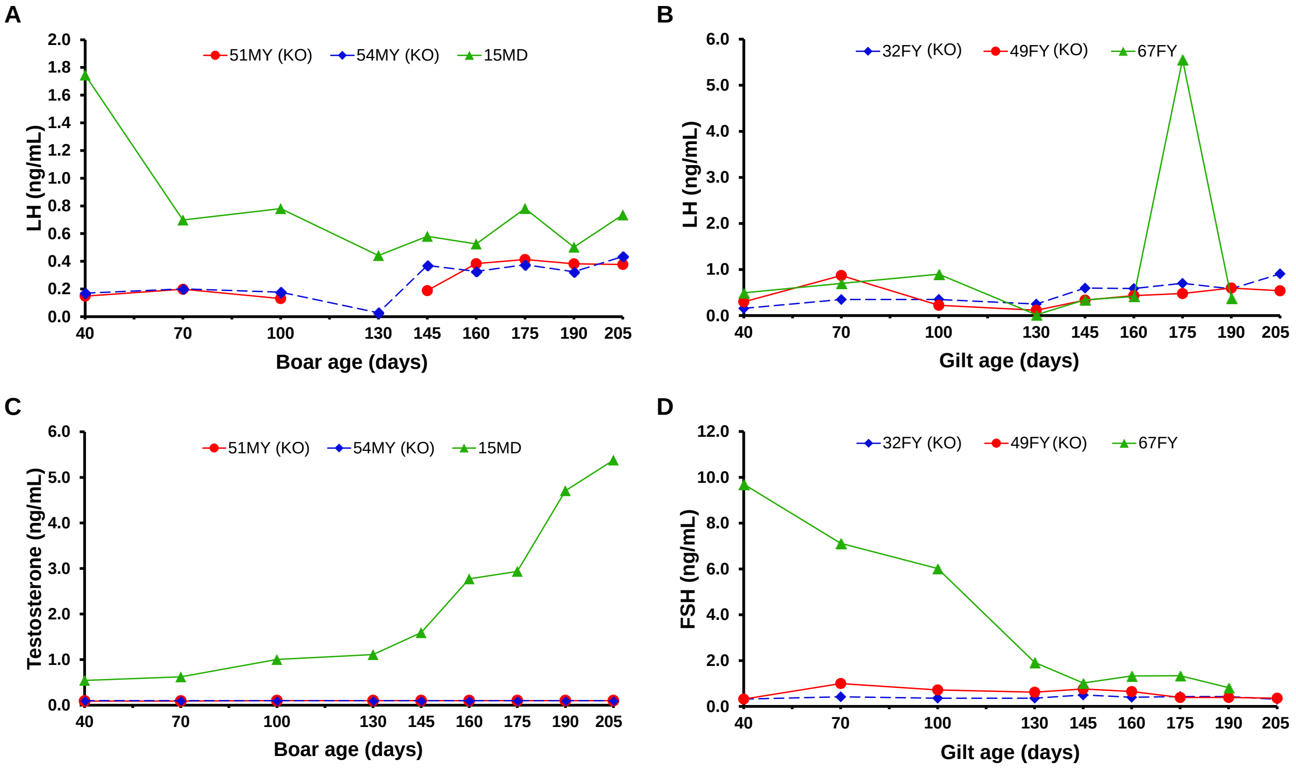

Supplementary Figure S3 | Individual hormone profiles of pigs with *KISS1*-disruptive editing percent >90%. **(A,B)** Serum LH concentrations sampled in boars **(A)** and gilts **(B)** across development. **(C)** Serum levels of testosterone up to 205 days of age in boars. **(D)** Concentration of serum FSH in mosaic gilts during development. The boar 15MD was estimated a *KISS1*-disruptive allele frequency of 88% after resequencing.
